# Reduced influence of perceptual context in mild traumatic brain injury is not an illusion

**DOI:** 10.1038/s41598-024-56713-y

**Published:** 2024-03-18

**Authors:** A. Sidhu, L. Uiga, B. Langley, R. S. W. Masters

**Affiliations:** 1 Te Huataki Waiora School of Health, University of Waikato, Hamilton, 3240 New Zealand; 2 Department of Sport and Exercise Sciences, Manchester Metropolitan University, Manchester, UK

**Keywords:** Psychology, Perception

## Abstract

Perceptual grouping is impaired following mild traumatic brain injury (mTBI). This may affect visual size perception, a process influenced by perceptual grouping abilities. We conducted two experiments to evaluate visual size perception in people with self-reported history of mTBI, using two different size-contrast illusions: the Ebbinghaus Illusion (Experiment 1) and the Müller-Lyer illusion (Experiment 2). In Experiment 1, individuals with mTBI and healthy controls were asked to compare the size of two target circles that were either the same size or different sizes. The target circles appeared by themselves (no-context condition), or were surrounded by smaller or larger circles (context condition). Similar levels of accuracy were evident between the groups in the no-context condition. However, size judgements by mTBI participants were more accurate in the context condition, suggesting that they processed the target circles separately from the surrounding circles. In Experiment 2, individuals with mTBI and healthy controls judged the length of parallel lines that appeared with arrowheads (context condition) or without arrowheads (no context condition). Consistent with Experiment 1, size judgements by mTBI participants were more accurate than size judgements by control participants in the context condition. These findings suggest that mTBI influences size perception by impairing perceptual grouping of visual stimuli in near proximity.

## Introduction

Mild traumatic brain injury (mTBI), also commonly known as concussion, is a major public health concern affecting 42 million people worldwide each year^1^. mTBI is the result of external biomechanical forces (accelerative-decelerative forces) transmitted to the head, neck, or body, either through direct impact (e.g. head-to-head collisions) or indirect rotational forces (e.g. whiplash type injuries from tackling or a car accident)^2,3^. These biomechanical forces can induce tensile and shearing stress on the axonal fibers within the white matter tracts, resulting in diffuse axonal injury ^4^, which in turn can initiate a neurometabolic cascade^5,6^ of neurochemical, ionic, and metabolic changes that disrupt normal communication within the brain^7–10^.

Given that approximately half of the brain’s neural pathways are dedicated to vision, the visual system is particularly vulnerable to shearing injury following mTBI^11^. As a result, considerable attention has been drawn to a variety of visual impairments associated with mild traumatic brain injury (mTBI)^12–14^, including defects in visual acuity^15^, blurred vision^12^, deficits in accommodation^16^ and binocular disparity^17^. However, little attention has been given to the perception of visual stimuli or the integration of visual information following mTBI.

Physiologically, the process of integrating individual elements in a visual image into a unified percept, a concept known as perceptual organization, is mediated by horizontal connections in the early visual cortex (V1)^18^. These horizontal connections, formed by the axon collaterals of pyramidal neurons in layer 2/3 of the visual cortex, run parallel to the surface of the brain. They connect neurons with similar response characteristics, such as preferred orientation, and play a crucial role in integrating information from separate parts of the visual field. More specifically, the V1 receives direct input of visual information from the environment and modulates it through horizontal connections in a feedforward fashion from low level visual areas to higher level areas^18^. Such organization allows the perception of visual stimuli to be context-specific, whereby the neural response to a visual stimulus depends on the surrounding stimuli or background^19^. For instance, when perceiving and judging attributes such as size, location or orientation of an object, the visual system often automatically takes into account surrounding elements in the visual scene^20^. This integration of visual information functions in accordance with the Gestalt grouping principles^21–24^. The Gestalt principles refer to specific ways in which humans automatically and holistically group visual features into coherent, whole objects and backgrounds^25^. The principles include grouping by proximity, similarity, good continuation, common fate and connectedness^23^.

It has been proposed that individuals with mTBI may demonstrate deficits in perceptual organization^26^. For instance, individuals with mTBI are reported to perform worse than controls on tasks that rely on grouping local elements into global wholes, such as perception of glass patterns^27^ and contour integration^26^. However, a limited number of studies have examined the principles of perceptual organization following mTBI, emphasizing the need for further research in this area to gain a deeper understanding of the mechanisms underlying visual perception in this population.

The aim of the present study was to examine perceptual organizational abilities in mTBI using Gestalt grouping principles. Of the five Gestalts principles, the law of proximity is considered one of the most important grouping principles of perceptual organization^28^. The principle of proximity states that when elements are in close proximity to one another, they tend to be perceived as single, cohesive group^23,24,29^. A well-known example of grouping by proximity is size-contrast illusions (e.g. Ebbinghaus illusion, Müller-Lyer), in which the appearance of a target stimulus (e.g. length, width or diameter) is distorted by the surrounding or attached context (e.g. Refs.^30–32^). For instance, in the Ebbinghaus illusion the perceived size of a central target is significantly altered by the sizes of a set of surrounding circles (i.e. appears smaller when surrounded by larger circles, appears larger when surrounded by smaller circles) because healthy individuals tend to process the size of the target circle in relation to the surrounding circles. A similar effect can be observed in the Müller-Lyer illusion, where the perceived length of a line is influenced by the orientation of the arrowheads attached to it. Specifically, when the arrowheads point inwards, the line appears longer compared to when the arrowheads point outwards^33^. This effect is argued to be caused by the closer spatial proximity of the inward-pointing arrowheads to the shaft^33^, as individuals often focus on comparing the size of the whole figure rather than the length of the two lines^34^. In other words, healthy individuals exhibit a globally oriented perceptual style when perceiving neighbouring objects in their visual field^35^. Conversely, individuals with a deficit in perceptual grouping (i.e. unable to group individual features into a whole) display a more locally oriented perceptual style^36,37^. This results in reduced susceptibility to visual illusions as a consequence of focusing only on the target and the parts of the inducers that are located in its close proximity^30,38,39^.

Thus, the study of size-contrast illusions is of strong heuristic value when examining perceptual grouping abilities in individuals with mTBI. To the best of our knowledge, visual illusion susceptibility has yet to be examined in mTBI. As a potential method for determining the extent to which perceptual organization is impaired in mTBI, we investigated whether individuals who had sustained a recent mTBI use the Gestalt principle of proximity when viewing size-contrast illusions. We hypothesized that if individuals with mTBI have impaired perceptual grouping abilities’, as suggested by atypical perceptual organization^26^, then they might demonstrate decreased susceptibility to size-contrast illusions than controls (i.e. more accurate at size judgements). Conversely, if individuals with mTBI do use the Gestalt principle of proximity, we would expect them to be susceptible to size-contrast illusions. To test this hypothesis, we conducted two laboratory experiments in which we asked people with and without self-reported mTBI to make perceptual judgments of the Ebbinghaus illusion (Experiment 1) and the Müller-Lyer illusion (Experiment 2).

## Experiment 1

In Experiment 1, we investigated the effects of surrounding context on size perception using the Ebbinghaus illusion in individuals with mTBI compared to healthy controls. The Ebbinghaus illusion task assesses how individuals’ size perception is sensitive to contextual features^40,41^. In a series of forced-choice judgments, participants were asked to determine whether two ‘target’ circles presented side by side on a computer screen were the same size or different. In the context condition, the target circle was surrounded by smaller or larger circles that created a context with potential to alter discrimination of its size.

### Methods

#### Participants

An opportunistic convenience sampling approach was used to recruit participants through flyers advertised on several social media platforms (e.g. Facebook, Twitter) and university-based email lists of students and staff. To be included in the mTBI group, participants were required to self-report ≥ 1 head injury within the last 12 months (see Sect. "Assessment of mTBI history" for assessment of mTBI history). To be included in the control group, participants were required to self-report no prior history of head injury. Individuals using psychoactive medications or with psychiatric conditions, were excluded from the experiment. Of those who expressed an interest in participating in the study, 84 were eligible, including 24 people with mTBI (14 females, 10 males; mean age = 18.75 years, SD = 1.39 years) and 60 healthy controls (30 females, 30 males; mean age = 19.98 years, SD = 5.42 years).

All participants had normal or corrected-to-normal vision. The experimental protocol was approved by the University of Waikato Ethics Committee and all participants provided informed consent electronically. Participants completed the experiment online and were advised that they were free to withdraw from the experiment at any time by closing the browser. There was no compensation provided for study participation.

#### Assessment of mTBI history

Detailed mTBI history was ascertained using the Ohio State University Traumatic Brain Injury Identification Method (OSU TBI-ID), a standardized procedure to elicit the lifetime history of TBI^42,43^. The measure consists of 11 items that measure the presence of historical TBI, cause of TBI, length of loss of consciousness and age of first and last occurrence of loss of consciousness. Participants in this experiment completed an online, self-administered version of the OSU TBI-ID, previously validated by Lequerica et al.^44^.

#### Apparatus and stimuli

**Figure 1:**
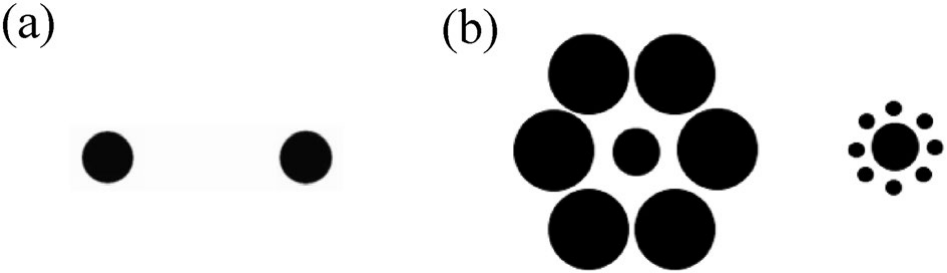
Examples of the stimuli used in the no context condition (Panel (**a**)) and the context condition (Panel (**b**)). In both conditions, the target circles are the same size. However, in the context condition (Panel (**b**)), the target circle on the left typically appears smaller than the target circle on the right (Ebbinghaus illusion).

The experiment was created using the OSWeb extension of OpenSesame^45^, an experiment builder available online (https://osdoc.cogsci.nl/). Each stimulus was prepared on a PowerPoint slide and then uploaded onto the software in order to “fix” the image sizes so that the stimulus was always displayed with the same size regardless of the browser window (and screen size). The experiment was then imported into a JATOS (Just Another Tool for Online Studies)^46^ server, where it was hosted and the data was stored. Participants were provided a link to the experiment and were instructed to use their personal laptop or computer. Participants were asked to sit up straight and position themselves at arm's length from the screen (51 cm); however, it is important to note that this may have resulted in variations in viewing difference due to differing compliance (and armlength) of the participants. In each stimulus slide, two white target circles were presented in horizontal orientation on a black background. The target circles appeared either with surrounding inducer circles (context condition) or without surrounding inducer circles (no context condition) (see Fig. 1). The center-to-center distance between the targets was 2.2 cm. In the context condition, one target circle was surrounded by 6 larger inducers (2.5 cm in diameter), whereas the other target circle was surrounded by 8 smaller inducers (0.3 cm in diameter). The side of the stimulus slide on which the smaller and larger surrounding inducers appeared was counterbalanced. In both the context and no context conditions, the target circles were either identical in size (same size condition) or different in size (different size condition) and ranged in size from 1.3, 1.5 or 1.7 cm. The position of the differently sized target circles (left / right) was counterbalanced in the different size condition. In the same size condition, there were 6 combinations: 3 size pairs (1.3–1.3/1.5–1.5/1.7–1.7) × 2 target-inducer pairs (large-small/small-large), and in the context different size condition, there were 8 combinations: 2 size pairs (1.3–1.5/1.5–1.7) × 2 target-inducer pairs (large-small/small-large) × 2 positions (left/right). Participants completed both context conditions twice, thus completing a total of 28 trials with context. In the no context same size condition, there were 3 combinations: 3 size pairs (1.3–1.3/1.5–1.5/1.7–1.7), and in the no context different size condition, there were 4 combinations: 2 size pairs (1.3–1.5/1.5–1.7) × 2 position (left/right). Participants completed the no context condition four times, in order to match the number of trials in the context condition. Each stimulus slide remained on the screen until the participant responded (or for a maximum of 3 s). In sum, participants completed 56 trials of the side-by-side comparison task (28 trials with context, 28 trials without context). Trials were presented in a randomized order via a JATOS randomisation algorithm.

#### Measures

A two-alternative forced choice paradigm was used, in which the participant had to decide whether the two target circles appeared the same or different in size. Participants recorded their answer by pressing the “A” key (same size) or the “L” key (different size) on the keyboard. The participants were not informed if an answer was correct or incorrect.

#### Procedure

Participants opened an online link, which directed them to a participant information sheet containing all study information and an electronic consent form. After providing consent, participants completed the Ohio State University Traumatic Brain Injury Identification Method form, the Rivermead Post Concussion questionnaire, and a general health questionnaire. All measures were self-reported online using the Qualtrics survey platform. Upon completion of the questionnaires, participants were then automatically linked to the experimental study, where they completed 6 practice trials followed by 56 experimental trials, during which they were instructed to indicate whether the circles that appeared on the screen were the same size (press the “A” key on the keyboard) or a different size (press the “L” key on the keyboard). The whole procedure took approximately 15 min. All methods were performed in accordance with the relevant guidelines and regulations.

#### Statistical analysis

Accuracy was calculated by averaging the percentage of correct responses for each condition in each group of participants. Shapiro-Wilks test was used to test for normality (α = 0.05). Due to unequal sample sizes and a non-normal distribution (*p* < 0.05), we employed the Aligned Rank Transform (ART) procedure using the ARTool package in R (version 4.0.2)^47^. Unlike conventional nonparametric statistical tests, which are limited to analyzing a single factor at a time, the ART procedure allows the simultaneous examination of multiple factors. ART involves a preprocessing step that aligns all the responses for possible main effects and interactions, before assigning averaged ranks to the aligned responses. The resulting aligned and rank data can then be analysed using standard ANOVA techniques.

In our study, a two-way repeated-measures ANOVA was carried out to examine the interaction between Condition (context and no context) and Group (mTBI and controls) following the ART procedure. Post hoc pairwise comparisons were carried out using an additional align and rank procedure called Aligned Rank Transform Contrasts (ART-C) with Holm-Bonferroni corrections, because they have been shown to have more statistical power than other such tests (e.g. *t *test, Mann–Whitney *U* test, Wilcoxon signed-rank test), and do not inflate Type I error rates^48^.

### Results

**Figure 2:**
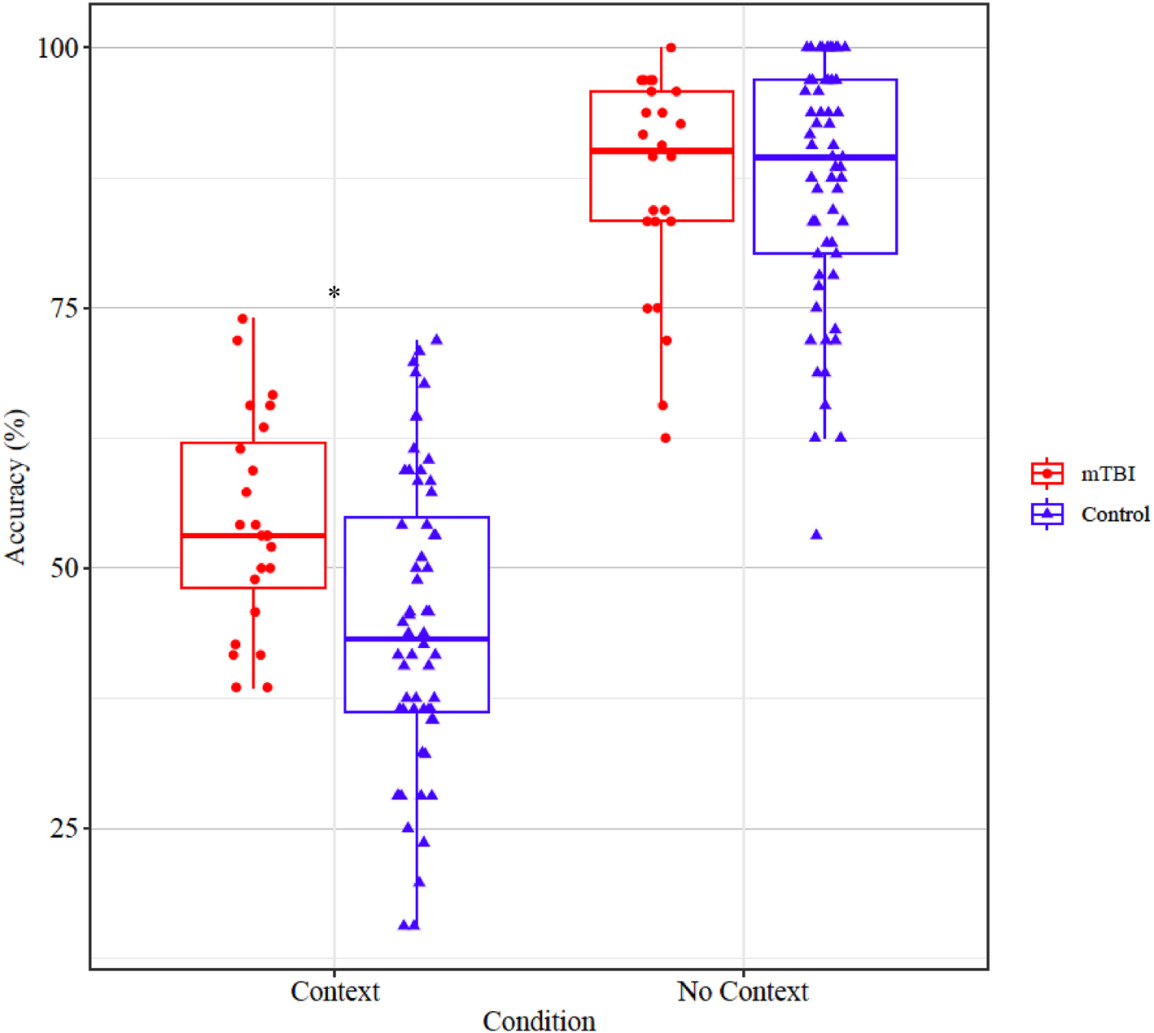
Scatter box-plot displaying accuracy scores (percentage) of mTBI and control participants in the “Context” and “No Context” conditions. The boxes represent the interquartile range (25–75th percentile), with the median indicated by the horizontal line within the box. The scattered points around each box represent the accuracy scores of individual participants. Significant differences between groups are marked with (*) for *p* < 0.01.

Statistically significant main effects were evident for Group, *F*(1,81) = 7.083, *p* < 0.001, and Condition, *F*(1,81) = 357.53, *p* < 0.001. A two-way interaction was evident between Group and Condition, *F*(1,81) = 4.936, *p* = 0.029. Post-hoc pairwise comparisons revealed no statistically significant difference in judgement accuracy between the mTBI group and the control group in the no context condition (t = − 0.093, *p* = 0.926). However, a significant difference was evident between the mTBI group and the control group in the context condition, (t = 3.045, *p* = 0.005). Figure 2 displays the distribution of accuracy scores in each group (mTBI/control) as a function of condition (context/no context).

### Discussion

The two groups demonstrated a similar percentage of correct responses in the no-context condition. By contrast, the mTBI group exhibited significantly reduced susceptibility to the Ebbinghaus illusion, performing more accurately than controls in the context condition. These results suggest that people with mTBI may have more difficulty perceptually grouping surrounding context in their visual field. However, in order to better understand the nature of reduced contextual integration in people with mTBI, the use of another size-contrast illusion is necessary. Therefore, in Experiment 2, we tested whether perceptual grouping abilities in people with mTBI differ when presented with the Müller-Lyer illusion.

## Experiment 2

In Experiment 2, the effects of surrounding context on size perception were investigated using the Müller-Lyer illusion in individuals with mTBI compared to healthy controls. The Müller-Lyer illusion consists of a set of parallel lines with opposing arrowheads on each end. When the arrowheads point outward, the line is generally perceived to be longer than when the arrowheads point inward. Consistent with the Ebbinghaus illusion, the Müller-Lyer illusion occurs because perception of the length of the lines is influenced by the attached visual context. In a series of forced-choice judgments, participants were asked to determine whether two lines presented parallel to each other on a computer screen were the same size or different.

### Methods

#### Participants

Participants were recruited in the same manner as Experiment 1. A total of 60 people expressed an interest in participating in the study. Participants included 18 individuals with mTBI (7 females, 11 males; mean age = 19.23 years, SD = 2.13 years), and 42 healthy controls (15 females, 27 males; mean age = 18.78 years, SD = 1.19 years). Inclusion criteria were identical to Experiment 1 (see Sect. "Methods"). The experimental protocol was approved by the University of Waikato Ethics Committee. Participants provided informed consent electronically and were advised that they were free to withdraw from the experiment at any time by closing the browser.

#### Assessment of mTBI history

Assessment of mTBI history was identical to Experiment 1.

#### Apparatus and stimuli

**Figure 3:**
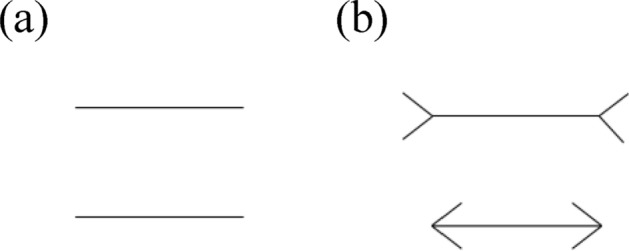
Example images of the two parallel lines in the no context condition (Panel (**a**)) and the context condition (Panel (**b**)). In both conditions, the parallel lines are equivalent in length. However, in the context condition (Panel (**b**)), perception of the length of the line is distorted by the presence of outward or inward pointing arrowheads at the ends of the line. The upper line is typically perceived to be longer than the bottom line (Müller-Lyer illusion).

As in Experiment 1, the OSWeb extension of OpenSesame^45^ was used to build the experiment and participants were instructed to use their personal laptop or computer. In each stimulus slide, two parallel (white) lines were presented in horizontal orientation on a black background. The parallel lines appeared either with arrowheads attached at the ends of the line (context condition) or without arrowheads attached (no context condition) (see Fig. 3). In the context condition, one line had inward-pointing arrows (> <) located at either end, and the other line had outward-pointing arrows at either end (< >). The horizontal line (top or bottom) on which the inward and outward-pointing arrows appeared was counterbalanced. In both the context and no context conditions, the parallel lines were either identical (same size condition) or different in length (different size condition) and ranged in length from 2.0, 2.5 or 3.0 cm. The distance between the two comparison lines was 1 cm. The position of each line in the different size condition was counterbalanced. In the context same size condition, there were 6 combinations: 3 size pairs (2.0–2.0/2.5–2.5/3.0–3.0) × 2 line-arrow pairs (inward-outward/outward-inward), and in the context different size condition, there were 8 combinations: 2 size pairs (2.0–2.5/2.5–3.0) × 2 line-arrow pairs (inward-outward/outward-inward) × 2 positions (top/bottom). Participants completed both context conditions twice, and thus performed a total of 28 trials with context. In the no context same size condition, there were 3 combinations: 3 size pairs (2.0–2.0/2.5–2.5/3.0–3.0), and in the no context different size condition, there were 4 combinations: 2 size pairs (2.0–2.5/2.5–3.0) × 2 positions (top/bottom). Participants completed both no context conditions twice. Each stimulus slide remained on the screen until the participant responded (or for a maximum of 3 s). In sum, participants completed 42 trials of the top–bottom comparison task (28 trials with context, 14 trials without context). Trials were presented in a randomized order via a JATOS randomisation algorithm.

#### Measures

The measures collected were the same as those in Experiment 1.

#### Procedure

The general procedure for the main experiment was identical to Experiment 1 (see Sect. "Procedure"). All methods were performed in accordance with the relevant guidelines and regulations.

#### Statistical analysis

Statistical analysis was identical to Experiment 1.

### Results

**Figure 4:**
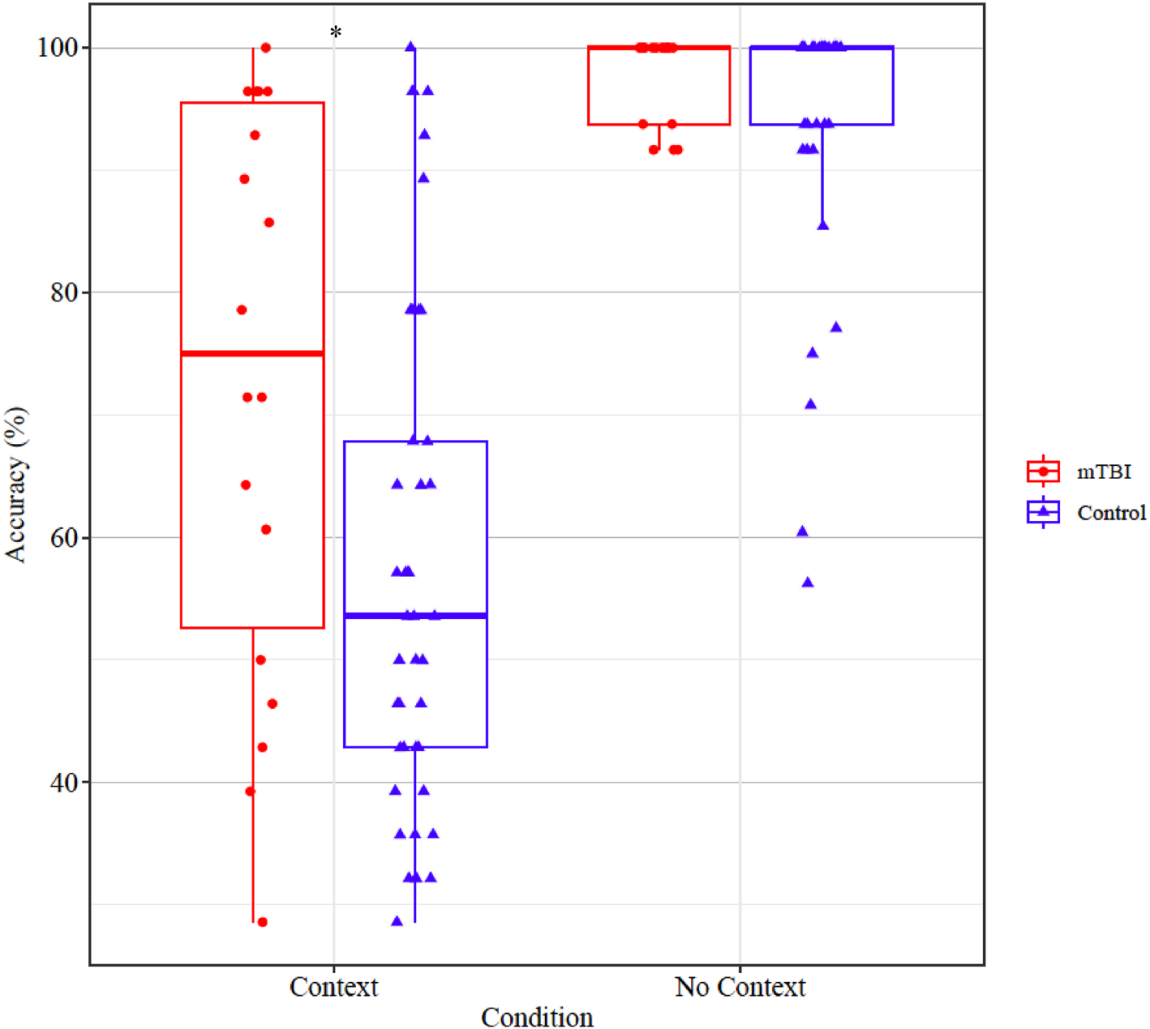
Scatter box-plot displaying accuracy scores (percentage) of mTBI and control participants in the “Context” and “No Context” conditions. The boxes represent the interquartile range (25–75th percentile), with the median indicated by the horizontal line within the box. The scattered points around each box represent the accuracy scores of individual participants. Significant differences between groups are marked with (*) for *p* < 0.05.

Statistically significant main effects were evident for Group, *F*(1,58) = 14.519, *p* < 0.001, and Condition, *F*(1,58) = 133.925, *p* < 0.001. A two-way interaction was evident between Group and Condition, *F*(1,58) = 11.488, *p* < 0.001. Post-hoc pairwise comparisons revealed no statistically significant difference in judgement accuracy between the mTBI group and the control group in the no context condition (t = 0.711, *p* = 0.478). However, a significant difference was evident between the mTBI group and the control group in the context condition (t = 2.562, *p* = 0.023). Figure 4 displays the distribution of accuracy scores in each group (mTBI/control) as a function of condition (context/no context).

### Discussion

In keeping with the findings in Experiment 1, participants in the mTBI group displayed more accurate size judgments than participants in the control group in the context condition, suggesting that those with mTBI were less susceptible to the Müller-Lyer illusion than participants without mTBI. This experiment provides further evidence that mTBI influences size perception by impairing perceptual grouping of visual stimuli in near proximity.

## General discussion

In two experiments, different size-contrast illusions (i.e. the Ebbinghaus illusion and the Müller-Lyer illusion) were used to examine visual size perception in participants with or without self-reported symptoms of mTBI. Participants who reported symptoms of mTBI were found to display significantly better size-contrast judgments for both illusions, implying that they were less susceptible to the perceptual distortion that is typically caused by the illusion. These results suggest that participants in the mTBI group may be less likely to perceptually group the target stimulus with the nearby visual elements (e.g. context) in comparison to controls. Together, our results provide support for the hypothesis that individuals with mTBI have reduced ability to automatically group by proximity as a result of impaired perceptual organization of the visual field (i.e. processing of individual features of a visual stimuli). Intact perceptual grouping processes would have led to automatic grouping of the surrounding context used in each experiment, resulting in a normal visual illusion effect as observed in the control group.

Previous studies that have used size-contrast illusions have hypothesized that the grouping of contextual information is mediated by long-range connections between neurons of similar orientated stimuli (i.e. edges and lines) within the same visual area^49^. These long-range interactions can be related to the intrinsic horizontal connections of the visual cortex, which provide a substrate for integrating information between cortical areas to produce coherent perception^49^. However, with weakened or disrupted connections, visual perception becomes more local and less affected by the surrounding context^50^. For example, Káldy and Kovács^51^ found that four-year-old children performed more accurately in a visual size discrimination task than adults. The authors argued that the superior performance of young children may be attributed to the incomplete maturation of neural circuits that facilitate the integration of contextual information. It is possible that this may be a similar case for individuals with mTBI; that is, rotational forces induced during mTBI damage long-range connections, which results in reduced cortical integration across visual fields^13,52^. This raises the possibility that failures to form context-based interactions may lead to the same sort of global processing differences that are found in young children whose visual systems have not yet fully developed the circuitry to support long-range connectivity.

Cortical integration is mediated by a plexus of long-range connections that allow neurons to coordinate information across cortical regions^53^. The mechanisms involved in perceptual grouping consist of long-range horizontal connections among neurons in layers 2/3 of V1 and V2^54^, which facilitate center-surround interactions within the receptive fields, enable groupings of collinear oriented elements and contribute to early object formation^55,56^. Visual information is then relayed to ventral cortical areas such as V4 (extrastriate area), where it is further integrated into more complex representations of visual stimuli^54–57^. Researchers have argued that mTBI can disrupt cortical processing, which may lead to impaired connections for cortical integration, making perceptual grouping, a process that integrates and coordinates information, particularly susceptible to disruption of cortical integration following mTBI^52^.

In the present study, our findings suggest that mTBI influences size perception by impairing perceptual grouping of visual stimuli in near proximity. This finding may help to explain why individuals with mTBI tend to perform poorly on visual perception tasks that rely on grouping multiple stimulus elements within the visual field; that is, when the ability to organize visual stimuli into meaningful patterns or structures is crucial for accurate perception. For example, one study showed that a small sample of adult patients with mTBI (*n* = 11) displayed impaired ability to discriminate between glass patterns (i.e. visual stimuli made from multiple pairs of dots, that give the percept of a coherent pattern), thought to be caused by widespread disruption in visual processing that extends to both major extra-striate visual cortex processing streams after axonal disruption^27^. Costa et al.^26^ also reported that individuals with mTBI performed significantly worse than controls in contour integration, suggesting a decreased capacity to integrate collinear Gabor elements into global shapes. The authors argued that the decline in performance might stem from diffuse axonal injury. Since this form of injury involves widespread damage to the white matter tracts, it could be speculated that the ventral visual stream necessary for the recognition of objects may be impacted. Therefore, it is postulated that individuals with mTBI may exhibit impairments in effectively processing perceptual grouping cues (i.e. surrounding or attached contextual stimuli), which may enable them to perceive the individual features (i.e. the details) of a visual stimulus first, potentially leading to a reduction in higher-order visual functions (i.e. recognizing shapes and objects).

Our study has a number of limitations and its results should be interpreted with caution. First, the online opportunistic convenience sampling procedure that we adopted in both experiments resulted in unequal sample sizes, with a greater number of non-mTBI participants than mTBI participants. In both cases, the percentage of participants reporting symptoms of mTBI within the previous 12 months was approximately 33%. This is substantially greater than the percentage typically reported in the general population annually (e.g. 1.2%)^58,59^; however, it is likely that sampling bias resulted in participation by students with an interest in sport (esp. rugby, soccer, netball), who therefore may have been more likely to report symptoms of recent mTBI. Second, while the use of visual illusions provides a measure of how the brain perceptually groups visual information into a coherent percept, it is important to note that alternative explanations have been proposed regarding the perception of visual illusions. Some researchers have argued that reduced susceptibility to visual illusions could be due to weakened top-down processing (knowledge-driven processing, also known as predictive coding), which prevents higher-level information, such as context, from modulating initial perception^60,61^. In other words, perception of the illusion is based solely on the sensory inputs (i.e. the real image), without any influence from prior experiences or knowledge of physical attributes of the world. This argument is upheld in people with schizophrenia who appear not to utilize top-down processing and, thus, often demonstrate reduced susceptibility to illusions^62^. Therefore, it is possible that top-down processing could also be affected by mTBI and potentially cause abnormalities in the perception of visual illusions.

Third, the use of self-report rather than medical diagnosis of mTBI is potentially a concern due to several reasons. Self-reported measures are based on subjective information, and participants may have recall bias, which can impact the accuracy of the reported time since the injury, number of mTBI sustained, and the symptoms experienced. Furthermore, self-reported measures may not fully capture the extent and complexity of symptoms associated with mTBI, as some symptoms may go unreported due to lack of awareness or understanding.

Fourth, the online and cross-sectional design of the study limited our ability to examine individuals directly after sustaining an mTBI. Thus, we are unable to determine causality between mTBI and perceptual grouping. That is, other factors (i.e. recovery processes) associated with sustaining an mTBI may affect perceptual grouping mechanisms. This limitation is important to consider as it may not accurately reflect the acute stages of mTBI and may not be generalizable to the entire population of mTBI individuals. Therefore, a longitudinal study should be conducted to examine the evolution of perceptual grouping deficits in individuals with mTBI. This approach would involve initial assessment of perceptual grouping abilities immediately post-injury and repeated evaluations throughout the recovery period. Such studies would yield valuable insight into the nature and recovery of perceptual grouping deficits in mTBI.

Fifth, response times were not collected. Thus, we are unable to confirm whether the observed effects in the mTBI group are purely a perceptual phenomenon. Future studies should therefore consider incorporating response time indices to clarify whether increased accuracy in the mTBI group is related to slower response times.

In conclusion, the present findings provide evidence for impaired perceptual grouping of visual stimuli in near proximity among individuals with mTBI. This was reflected by better size discrimination accuracy in the context condition, which suggests that individuals with mTBI were less influenced by visual illusion effects because they were able to process the parts separately from the inducing context. That is, the mTBI participants may not have shown the same preference for global processing that has been found in healthy controls^35^; instead, they may have been more likely to process local features of the stimulus configuration first, as has been observed in people with autism^38^. However, to confirm the presence of a localized processing bias in individuals with mTBI, additional studies are necessary. Future work using local–global processing tasks such as the Navon task, could be utilized to examine how individuals with mTBI integrate and prioritize visual information. By manipulating the size and congruence of local and global features in visual stimuli, the Navon task can probe mechanisms underlying perceptual grouping, cognitive biases and attention allocation. It is further warranted to explore these behavioral measures in conjunction with eye-tracking, electrophysiological or functional imaging measurements, to gain insight into how individuals with mTBI scan visual scenes, prioritize different features or objects, and the neural mechanisms associated with visual perception. Last, given the heterogeneity of mTBI, future studies should consider replicating these experiments to test the generalizability of the findings.

## Data Availability

The datasets generated and/or analysed during the current study are available on OSF (https://osf.io/jcv7d/). All experiments methods were carried out in accordance with Scientific Reports guidelines and regulations.
